# A novel adaptive PD-type iterative learning control of the PMSM servo system with the friction uncertainty in low speeds

**DOI:** 10.1371/journal.pone.0279253

**Published:** 2023-01-18

**Authors:** Saleem Riaz, Rong Qi, Onder Tutsoy, Jamshed Iqbal

**Affiliations:** 1 School of Automation, Northwestern Polytechnical University, Shaanxi, Xi’an, PR China; 2 Department of Electrical-Electronics Engineering, Adana Alparslan Turkes Science and Technology University, Adana, Türkiye; 3 School of Computer Science, Faculty of Science and Engineering, University of Hull, Hull, United Kingdom; University of Bradford, UNITED KINGDOM

## Abstract

High precision demands in a large number of emerging robotic applications strengthened the role of the modern control laws in the position control of the Permanent Magnet Synchronous Motor (PMSM) servo system. This paper proposes a learning-based adaptive control approach to improve the PMSM position tracking in the presence of the friction uncertainty. In contrast to most of the reported works considering the servos operating at high speeds, this paper focuses on low speeds in which the friction stemmed deteriorations become more obvious. In this paper firstly, a servo model involving the Stribeck friction dynamics is formulated, and the unknown friction parameters are identified by a genetic algorithm from the offline data. Then, a feedforward controller is designed to inject the friction information into the loop and eliminate it before causing performance degradations. Since the friction is a kind of disturbance and leads to uncertainties having time-varying characters, an Adaptive Proportional Derivative (APD) type Iterative Learning Controller (ILC) named as the APD-ILC is designed to mitigate the friction effects. Finally, the proposed control approach is simulated in MATLAB/Simulink environment and it is compared with the conventional Proportional Integral Derivative (PID) controller, Proportional ILC (P-ILC), and Proportional Derivative ILC (PD-ILC) algorithms. The results confirm that the proposed APD-ILC significantly lessens the effects of the friction and thus noticeably improves the control performance in the low speeds of the PMSM.

## 1. Introduction

Recent emerging advancements in the automation technology have redefined the role of the robotics and mechatronics systems in modern-day industrial world. To perform various tasks like the welding, machine tending, grinding, packaging, assembling and material transporting, a variety of robots such as the industrial robot manipulators [1], wheeled mobile robots [2] and track-driven mobile robots [3] have been widely considered. Owing to stringent requirements of the precision and accuracy in a highly nonlinear and dynamic environments, there is an increasing demand to develop novel actuation and control technologies for these industrial robots [4, 5].

The PMSM has been broadly used in industrial robots, high-precision CNC machines, and other applications employing servo control due to its small size, simple structure, low moment of inertia, and high-power density. It is reported that the servo control systems performing repetitive tasks can theoretically achieve satisfactory tracking results using the ILC [6]. However, various disturbances, modeling errors [7], and time-varying system parameters [8] adversely affect the convergence of the ILC. Therefore, the control community is actively working on the ILC-based control of the PMSM servo systems working under the dynamic uncertainties [9]. Recently, researchers have combined the adaptive control and ILC to form Adaptive Iterative Learning Control (AILC) [10, 11], which exploits the ILC to solve the repetitive tracking problems [12, 13] and adaptive control to handle the system uncertainty problem. Thus, AILC improves the tracking accuracy of the PMSM servo system and also accelerates the controller speed of convergence in the presence of the uncertainties [14, 15].

Compared with the ‘traditional rotating motor and ball screw’ drive mode, a Permanent Magnet Linear Synchronous Motor (PMLSM) adopts a direct-drive mode, without any conversion links in the middle and offers larger thrust, low loss, and fast response. Owing to these advantages, the PMLSM has been widely preferred in a large number of industrial applications [16–18]. However, the parametric changes, friction, measurement disturbances, and cogging force affect the motor motion which cause formidable control challenges. This necessitates a robust controller design with an ability to accurately track the reference trajectory by eliminating the detrimental effects of the uncertainties [19, 20].

Due to the uncertainties in the PMSM servo system, its high precision tracking control with the conventional PID controller is challenging [21–23]. In addition, owing to the nature of underlying mathematical structure, it is not easy to fully compensate the nonlinearities of the motor with the linear control approaches having constant gains [24, 25]. Work reported in [26] expressed that a disturbance predictor-based control can improve the tracking accuracy of the controller since it gains the disturbance rejection capability. Another work [27] employed an iterative learning approach to reduce the controller position gain in order to enhance the position tracking of a linear motor working in specific state intervals. However, increase in the number of iterations reduced the controller convergence speed [28]. Similarly, an open-loop P-type ILC for the compensation of the measurement noise has been presented in [29]. The non-repetitive disturbance considerably fluctuated the motor position since the controller was unable to handle the instant random changes in the disturbance [30, 31].

Other types of control approaches without the friction disturbances are the intelligent control [32], adaptive fuzzy control [33] and Active Disturbance Rejection Control (ADRC) [34]. The Disturbance Observer (DOB) compensation methods are reviewed in [35]. The neural network control algorithm in [32] and the fuzzy control algorithm in [33, 36, 37] have approximated the friction characteristics by a genetic algorithm. A controller was then designed to compensate the friction. However, due to the hardware constraints, the intelligent control algorithm requires a large amount of online computations. The primary DOB disturbance compensation method in [38] and its counterpart ADRC in [34, 39] both suppressed the friction, but their dynamic performances and stability analyses were not carried out [40, 41].

In scientific literature, the PMSM vector control system has been established on the *dq*-axis transformed model with i_d_ = 0 in which the transformed *d*-axis current (i_d_) of the PMSM is kept at zero to ease the control problem. Generally, the influence of the uncertain factors such as the friction torque, cogging torque, modelling errors, and the time-varying parameters on the control system have been analyzed. The well-known state equations of the system have been established. Recently, a parametric AILC law has been formulated to lessens the deteriorations in the PMSM servo system’s tracking accuracy and error divergence caused by the model uncertainty. The primary method is to add adaptive iterative feature to the PD-based feedback control law. Through the parameter learning, the unknown parameters of the control law are identified. Then, based on the previous learning law, an enhanced version of the adaptive iterative learning law is presented. This corresponds to adding a time-domain estimate of the unknown parameters to the former learning law while taking full advantage of the information in both the continuous and discrete times.

Given the nonlinear approximation ability of the neural networks, a number of researchers have used them to identify the frictional torque and compensate it with the controller [42–45]. The neural network can be either used as the central controller or is combined with other control algorithms to gain adaptive control properties [46]. The robustness of the controller against the friction and other disturbances has been demonstrated in [47, 48] which present suitable entropy-based ILC compensation and in [49, 50] where various ILC approaches are introduced. Fuzzy control with adaptive internal model control can also be used as a disturbance suppression approach. For example, in [51, 52] an improved internal model control is utilized for the speed control and two disturbance observers estimate the moment of inertia and the PMSM load. In [51], the motor disturbances have observed by the combination of an uncertainty predictor and a state predictor. Its feedforward compensation has been applied to the control system by using the uncertainty predictor estimates which leads to a superior control performance. A further research reported in [53] presented a hybrid control scheme based on sliding mode control and disturbance rejection of a second order PMSM system whose efficiency has been demonstrated through experimental validation.

In recent years, a number of researchers have adopted robust adaptive control, especially the adaptive backstepping law, to achieve high accuracy in servo control [54–57]. Majority of the reported works considered the motor’s high speed and thus an improvement in performance at relatively low speeds is still an open research area. This paper focuses on the control of the PMSM running at low speeds where the friction Stribeck compensation problem occurs. To handle the identified friction uncertainty, the APD-ILC position control algorithm, which is an iterative and adaptive control approach, is developed. Simulations are performed to qualitatively analyze the results and verify the position tracking and the friction compensation abilities of the APD-ILC algorithm.

The control algorithm incorporates the friction model in the form of the feedforward control to generate a friction-free APD-ILC algorithm. The typical ILC relies on powerful mathematical calculations instead of precise models. On the other hand, the degree of function compensation is primarily determined by the filter’s bandwidth, which is restricted by variables such as the mechanical resonance in the system. Consequently, there is a limited capacity to adjust for friction nonlinearity at zero speed. Basically, the proposed technique updates the learning gain of the controller according to the magnitude of the error to improve the tracking accuracy of the system. At the same time, the exponential learning gain is introduced into the differential coefficient of the controller to speed up the convergence. Finally, the efficiency of the proposed control technique is demonstrated in simulation.

This article is divided into five sections. Section 2 derives the model of a PMSM, Section 3 provides the convergence analysis, theory of adaptive ILC, nonlinear gain function, and the sufficient conditions for the error convergence Section 4 presents simulation results to validate the proposed control approach and finally, Section 5 concludes the paper.

## 2. Permanent Magnet Synchronous Motor System (PMSM) model

This research considers a surface-mounted PMSM model transformed in the *dq*-axis. The typical PMSM mathematical model is given in Eq (1), which uses the notations presented in Table 1.

$$\{\begin{array}{c} \frac{di_{d}}{dt}=-\frac{R_{s}}{L_{d}}i_{d}+\frac{L_{q}}{L_{d}}n_{p}\omega i_{q}+\frac{1}{L_{d}}u_{d}\\ \frac{di_{q}}{dt}=-\frac{R_{s}}{L_{q}}i_{q}-\frac{L_{d}}{L_{q}}n_{p}\omega i_{d}-\frac{\psi_{f}}{L_{q}}n_{p}\omega +\frac{1}{L_{q}}u_{q}\\ \frac{d\omega }{dt}=-\frac{B_{m}}{J}\omega +\frac{n_{p}}{J}[\psi_{f}i_{q}+(L_{d}-L_{q})i_{d}i_{q}] \end{array}$$ (1)

where *u*_*d*_, *u*_*q*_ are the stator voltages and *i*_*d*_, *i*_*q*_ are the armature currents and the *L*_*d*_, *L*_*q*_ are the stator inductances on the *dq-*axis of the motor, *n*_*p*_ is the number of the pole pairs of the PMSM, *R*_*s*_ is the stator resistance, *ω* is the mechanical angular velocity of the motor rotor, *ψ*_*f*_ is the rotor flux corresponding to the permanent magnet, *J* is the rotational inertia, *B*_*m*_ is the viscous damping. In the actual system, to decouple the speed and current, the vector control mode of $i_{d}^{*}\equiv 0$ is often used. The equation of the electromagnetic torque can be written as:

$$T_{e}=J\frac{d\omega }{dt}+B_{m}\omega +T_{L}=K_{t}i_{q}$$ (2)

where *T*_*L*_ is the load torque, and *K*_*t*_ is the torque constant.

**Table 1:** Nomenclature.

Notation	Description
*T*_*e*_	Electromagnetic torque
*K*_*m*_	Equivalent gain of the PMSM driver
*u*(*t*)	Control input
*θ*	Motor angular position
*ω*	Motor angular speed
*J*_*m*_	Total inertia
*B*_*m*_	Damping coefficient
*T*_*L*_	Loading torque
*η*(*t*)	Random measurement noise
*x*(*t*)	System states
*A*,*B*,*C*,*E*,*D*	System vectors and matrices
*F*	System friction torque
*h*	Sampling time
*I*	Identity matrix with appropriate dimension
*y*_*d*_ = *θ*_*d*_(*t*)	Desired position trajectory
*k*	Current iteration index
*e*_*k*_	Tracking error
*y*_*k*_	System actual output
*K*_*P*_	Proportional gain
*K*_*D*_	Derivative gain
*u*_*k*_	Output of the APD-ILC controller
*K*_1_	ILC proportional learning gain
*K*_2_	ILC derivative learning gain
Δ*e*_*k*_	Sample differenced tracking error
*τ*_*P*_, *τ*_*D*_	Positive constant gains
*k*_1_	Upper bound of nonlinear function *f*(.)
*k*_0_	Lower bound of nonlinear function *f*(.)
*α*, *b*_*d*_, *b*_*η*_	Positive scaling numbers

Let *F* be the system friction torque, *θ* be the rotational angle of the motor rotor, *J* be the sum of the motor and load moment of inertia, then Eq (2) can be re-formulated as:

$$\{\begin{array}{c} J\frac{d\omega }{dt}=T_{e}-B_{m}\omega -T_{L}-F\\ \frac{d\omega }{dt}=\frac{1}{J}[K_{t}i_{q}-B_{m}\omega -T_{L}-F]\\ \frac{d\theta }{dt}=\omega \end{array}$$ (3)

A friction model in [58] is used here to fully describe the friction encountered by the PMSM servo system. The model is represented by the expression;

$$F=(F_{c}+(F_{s}-F_{c})e^{-(\dot{\theta }/{\dot{\theta }}_{s})})\mathrm{sgn}(\dot{\theta })+B_{m}\dot{\theta }$$ (4)

where *F*_*c*_ is the Coulomb friction torque, $\dot{\theta }$ is the rotational angular velocity, *F*_*c*_ is the static friction torque, ${\dot{\theta }}_{s}$ is the Stribeck characteristic velocity.

### 2.1 Detailed modelling of the Stribeck friction

**Fig 1:**
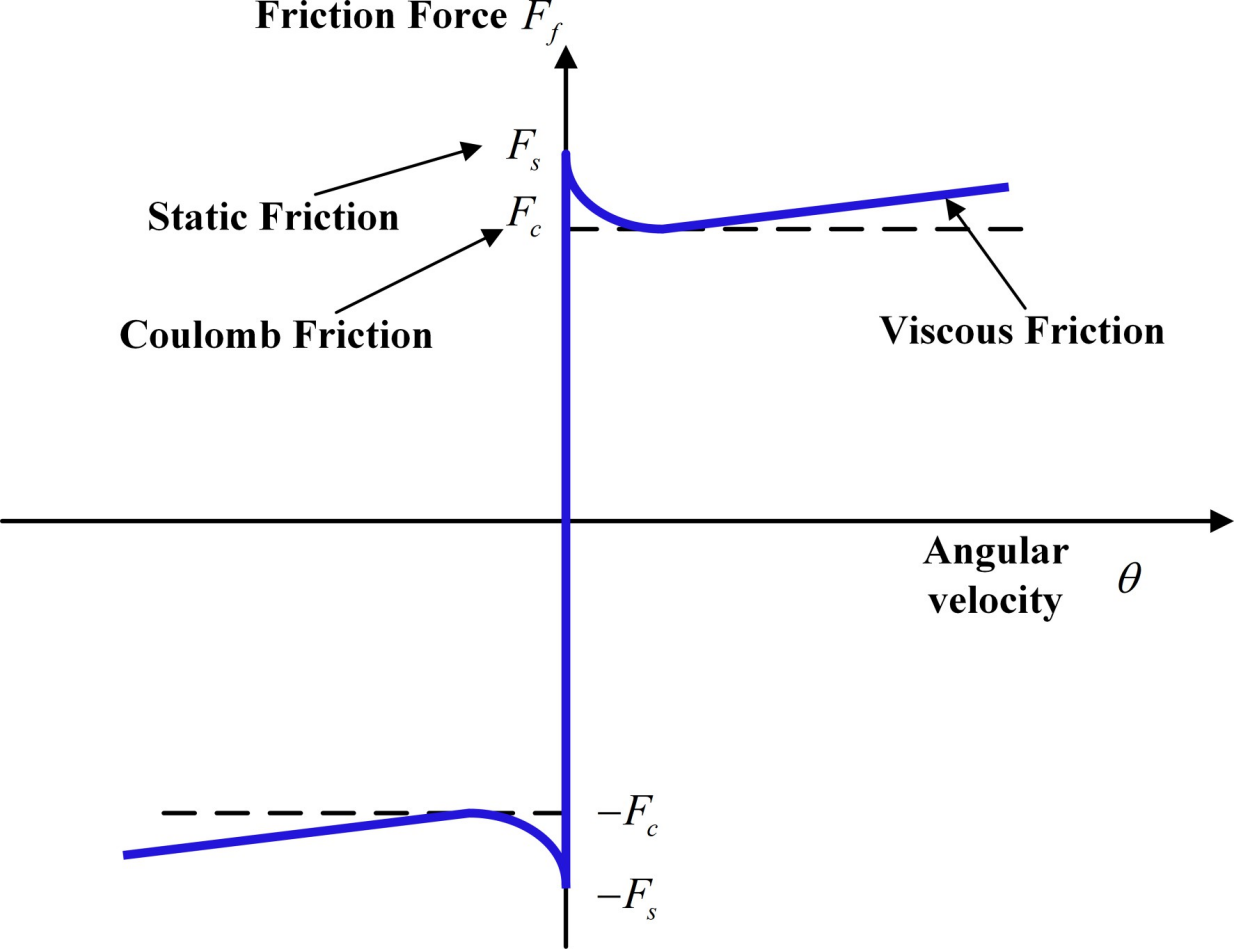
The Stribeck friction curve.

Stribeck curve in Fig 1 is a well-known tool for modelling of the friction. It essentially indicates the relationship between the friction force and angular velocity for different values of the friction. The Stribeck friction model can be expressed as in Eqs (5) and (6). When the static friction is $|\dot{\theta }(t)|<\alpha$, with *α* threshold, the dynamic friction *F*_*f*_(*t*) can be given by (5).


$$F_{f}(t)=\{\begin{array}{c} F_{m}\;F(t)>F_{m}\\ F(t)\;-F_{m}<F<F_{m}\\ -F_{m}\;F(t)<-F_{m} \end{array}$$ (5)

On the other hand, when the static friction is $|\dot{\theta }(t)|>\alpha$, *F*_*f*_(*t*) can be given as;

$$F_{f}(t)=(F_{c}+(F_{m}-F_{c})e^{-\alpha_{1}\dot{\theta }(t)})\mathrm{sgn}(\dot{\theta }(t))+B_{m}\dot{\theta }$$ (6)

where *F*_*m*_ denotes the driving force, *F*_*c*_ represents the maximum static friction force, *α*_1_ is a small scaling factor.

### 2.2 Identification of the friction parameters

**Fig 2:**
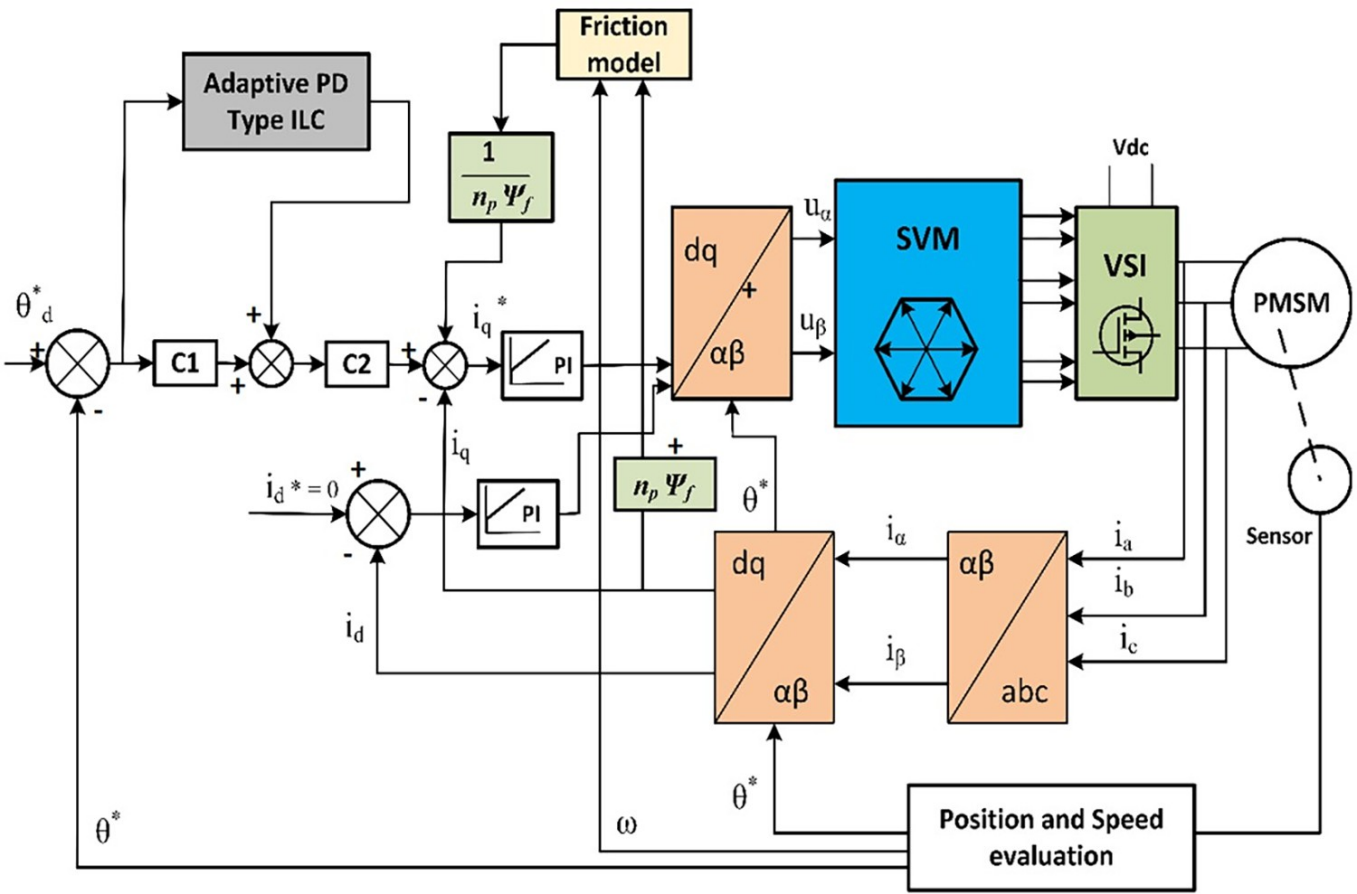
Overall control architecture.

The genetic algorithm is a probabilistic search optimization approach that imitates the organisms’ genetic and evolutionary processes in their natural environment. It can solve complex and poorly known nonlinear optimization problems without requiring the model of the system. The optimization search can be carried out throughout the range of a pre-determined unknown parameter space and the best parameter solutions are selected among them [59]. The genetic algorithm can also avoid local minima with rich and diverse exploration signals, so it gains a wide range of adaptability and strong robustness. Fig 2 shows the overall APD-ILC architecture.

In Fig 2, the desired angular position $\theta_{d}^{*}$ is compared with the measured angular position θ* to generate the angular position tracking error. This error is processed by the APD-ILC and the corresponding feedforward control signal is subtracted from the position tracking error weighted with the *C*_1_. This yields a further tracking error which is transformed by the *C*_2_ weighting value and compared with the *i*_*q*_ current on the *q-*axis together with the friction model output to produce the $i_{q}^{*}$ desired current. This comparison error is evaluated by the traditional PI controller which feeds the model transformed from *dq-*axis to *αβ*-axis. Similarly, in case of the desired current $i_{d}^{*}=0$, a further PI controller assesses the *i*_*d*_ current on the *d-*axis and provides the control signal for the *dq-*axis to *αβ*-axis transformer. The outputs of this transformer are modulated by the Space Vector Modulation (SVM) component and the Voltage Source Inverters (VSI) provides the required 3-phase voltage for the PMSM. When the system is in steady-state, the angular velocity becomes $\dot{\omega }=0$ and without the load *T*_*L*_ = 0, Eq (1) becomes *F* = *T*_*e*_ = *B*_*m*_*ω*.

In terms of the PMSM speed control, consider a set of constant speed *n*_*p*_*ψ*_*f*_ as the command signal. According to Eq (6), construct the identifier $\hat{F}(x,{\dot{\theta }}_{i})$ and set the parameter to be identified as

$$x={\left[F_{c},F_{s},\dot{\theta },B_{m}\right]}^{T}$$ (7)

Defining the identification error $e({\dot{\theta }}_{i},x)$ as;

$$e({\dot{\theta }}_{i},x)=\sqrt{\left[F({\dot{\theta }}_{i})-\hat{F}(x,{\dot{\theta }}_{i}\right)}{]}^{2}$$ (8)

where

$$F({\dot{\theta }}_{i})=(F_{c}+(F_{s}-F_{c})e^{-({\dot{\theta }}_{i}/{\dot{\theta }}_{i})})\mathrm{sgn}({\dot{\theta }}_{i})+B_{m}{\dot{\theta }}_{i}$$ (9)

A quadratic objective function is formulated as:

$$l=\frac{1}{2}\sum_{i=1}^{n}e{\left({\dot{\theta }}_{i},x\right)}^{2}$$ (10)

Typically, the traditional optimization methods begin randomly and then iteratively decide on the best result from the solution space. Due to the limited amount of information offered by a single set of training data, the search performance can be poor, and also the search may get locked in the local optimum solution thus entering in stall mode due to insufficient exploration. Properly excited search may help to steadily acquire the global optimal solution by preventing the optimization for superfluous spots and avoiding slipping into the local optimum solution.

Genetic algorithms include selection, crossover and mutation processes and offers a larger likelihood of the global convergence to the optimum solution and a greater capacity to solve the uncertain optimization problems. However, factors such as the crossover probability and mutation probability have an effect on the algorithm’s search results and efficiency thus highlighting critical role of the genetic algorithm parameters for a particular application is important.

### 2.3. Bit string mutation

The mutation of bit strings ensues through bit flips at random positions. For instance:

**Table:** 

1	0	1	0	0	1	0
				↓		
1	0	1	0	1	1	0

**Definition 1:** The *order* of the pattern is the number of 0’s and 1’s in the pattern and is denoted as *O*(*H*) (e.g. *O*(11*00**) = 4.

**Definition 2:** The *moment* of the pattern, i.e. the length of the pattern, is the distance from the left to right between the first non-* bit and the last non-* bit of the pattern. The moment of the pattern is denoted by *δ*(*H*). e.g.


δ(01**1)=3;δ(**0*1)=2;δ(***1**)=1

A random number of initial groups encoded with a certain length (which is proportional to the unknown parameter spaces) is created. Each individual is evaluated using the fitness function, and those with a better fitness rating are chosen to engage in the genetic operation, while those with a poor fitness score are removed from the solutions. A group of genetically modified individuals (through replication, crossover, or mutation) generates a new generation population until the stop requirement is fulfilled. The most fully developed person among the descendants is considered to be the outcome of the genetic algorithm. With the mutation, the sequence before the mutation *x* = *x*_1_*x*_2_…*x*_*i*_ might become to *x* = *x*_2_*x*_1_….*x*_*i*−2_ for *i* number of the initial population. The range of each population has a constrained value for each sample *k* between $\left[u_{\min}^{k},u_{\max}^{k}\right]$. The boundaries are implemented on the *x*_*k*_ parameter population as:

$$x_{k}=\{\begin{array}{c} u_{\min}^{k}\;\mathrm{if}\;random(0,1)=0\\ u_{\max}^{k}\;\mathrm{if}\;random(0,1)=1 \end{array}$$

where *random*(0,1) is generates 0 or 1 values randomly.

The problem of friction parameter identification is to find the parameter vector *x*in Eq (7) that minimizes the objective function in Eq (10). The decimal floating-point coding format is used for encoding the parameter vector to identify the individual solutions. The selection operation involves a random sampling method that saves the optimal individuals. The overall identified values are compared in Table 2 below. The crossover operation adopts the uniform crossover operator, and the mutation assumes the basic bit. The maximum evolutionary number is 200 with a population size *i* of 50. The crossover probability is 0.9 and the mutation probability is 0.05. Parameter range *F*_*s*_ is [0,1], *F*_*c*_ is [0,1], *B*_*m*_ is [0,0.05]. Finally, the Stribeck characteristic velocity is ${\dot{\theta }}_{s}=[0,0.05].$

**Table 2:** Friction identifier parameters.

	*F*_*c*_	*F*_*s*_	${\dot{\theta }}_{s}$	*B*_*m*_
Initial parameters	0.28	0.34	0.01	0.02
Identification results	0.2796	0.3885	0.0108	0.0206

## 3. Adaptive ILC-based feedforward controller

According to the Fig 1, the feedforward compensation is achieved in terms of manipulating the motor currents. Eq (2) can be re-written with the friction torque as:

$$J\frac{d\omega }{dt}=-B_{m}\omega +K_{t}(i_{q}-i_{F})-T_{L}$$ (11)

The friction torque *F* is given by

$$i_{F}=\frac{F}{K_{t}}$$ (12)

**Fig 3:**
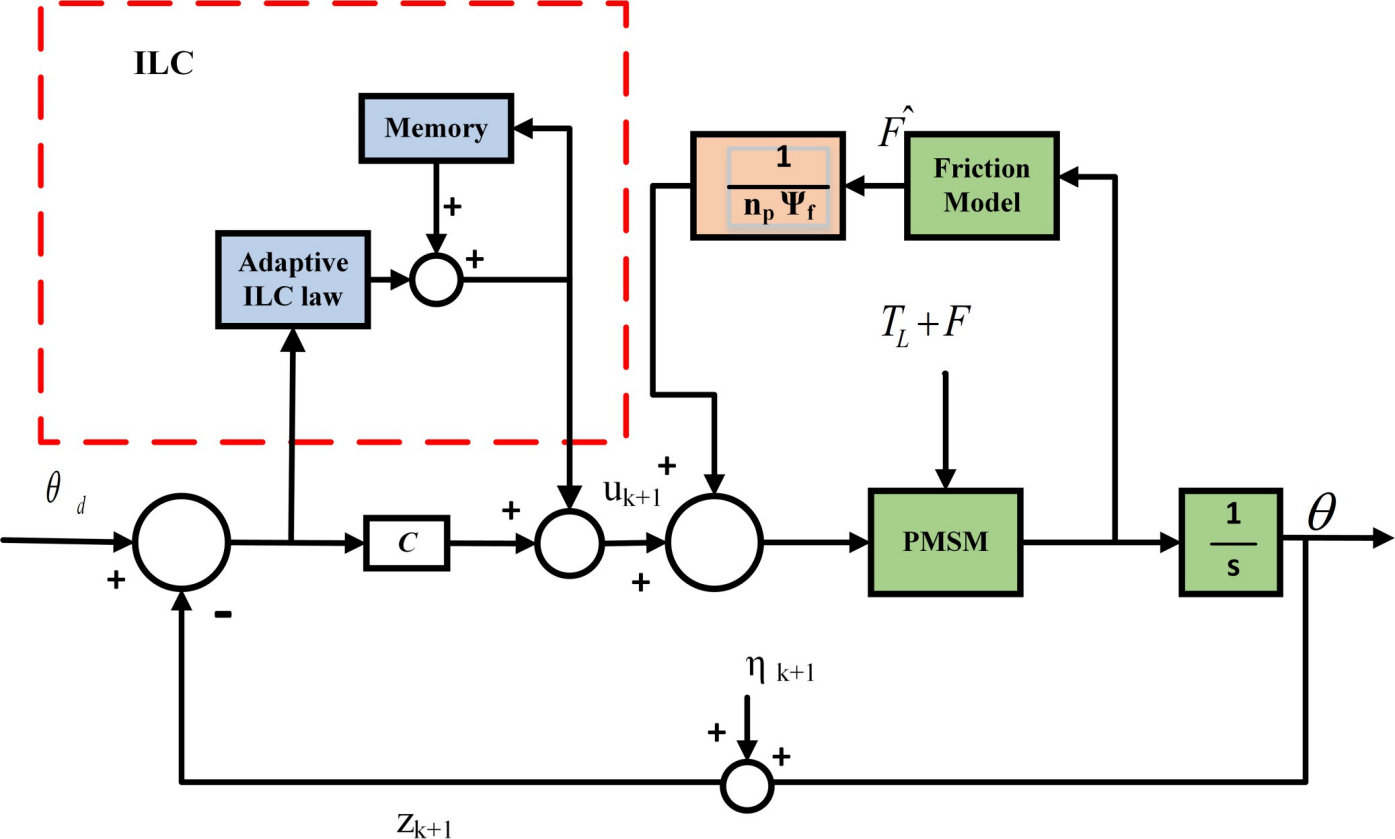
General block diagram illustrating the adaptive ILC-based feedforward controller.

The friction model identified in the section 2.2 is used to perform friction compensation of the PMSM servo system. The proposed feedforward controller is shown in Fig 2. The total control output of the system is sum of the outputs of the PI feedback controllers and the friction feedforward controller given by

$$i_{q}^{*}=i_{C}+i_{\hat{F}}$$ (13)

where $i_{\hat{F}}=\frac{\hat{F}}{K_{t}}$ is the estimated friction torque and *i*_*C*_ is produced by the adaptive ILC. The general control structure is shown in Fig 3.

The PMSM given in Eq (1) with the added Stribeck friction is linearized around the operating points and put in the state space form as:

$$\begin{array}{l} \underset{\dot{x}(t)}{\underset{︸}{\left[\begin{array}{c} \dot{\theta }\\ \dot{\omega } \end{array}\right]}}=\underset{A}{\underset{︸}{\left[\begin{array}{c} 0\;1\\ 0\;\frac{B_{m}}{J} \end{array}\right]}}\underset{x(t)}{\underset{︸}{\left[\begin{array}{c} \theta \\ \omega \end{array}\right]}}+\underset{B}{\underset{︸}{\left[\begin{array}{c} 0\\ \frac{K_{t}}{J} \end{array}\right]}}\underset{u(t)}{\underset{︸}{i_{q}}}+\underset{d(T_{L},T_{f})}{\underset{︸}{\left[\begin{array}{c} 0\\ -\frac{T_{L}+F}{J} \end{array}\right]}}\\ y(t)=\underset{C}{\underset{︸}{\left[\begin{array}{c} 1\;0 \end{array}\right]}}\underset{x(t)}{\underset{︸}{\left[\begin{array}{l} \theta \\ \omega \end{array}\right]}}+\eta (t) \end{array}$$ (14)

where *A*,*B*,*C* are the linear system matrices and vectors, *d*(*T*_*L*_,*T*_*F*_) is the disturbances stemmed from the load and the Stribeck friction, *x*(*t*) system state vector, *u*(*t*) is the controlled *i*_*q*_ current, *η*(*t*) is the measurement noise type uncertainty. Eq (15) presents the briefed version of the state space representation in Eq (14).


$$\{\begin{array}{l} \dot{x}(t)=Ax(t)+Bu(t)+d(T_{L},T_{f})\\ y(t)=Cx(t)+\eta_{t} \end{array}$$ (15)

The next sub-section introduces the P-ILC design.

### 3.1. P-ILC design

A simple control law based on P-ILC is expressed as:

$${\bar{u}}_{{}_{k+1}}(t)={\bar{u}}_{{}_{k}}(t)+K_{P}{\hat{e}}_{{}_{k}}(t)$$ (16)

where ${\bar{u}}_{{}_{k+1}}(t)$ represents the updated control signal and ${\hat{e}}_{k}(t)$ is the tracking error.

The well-known condition for the error convergence of the P-ILC algorithm can be described by:

$$\|I-K_{p}CB\|<1$$ (17)

The next sub-section provides the APD-ILC algorithm.

### 3.2. APD-ILC algorithm

Fig 3 presents the configuration of the APD-ILC algorithm applied to the PMSM with the friction uncertainty. In Fig 3, *y*_*d*_ denotes the desired position, *y*_*k*_ represents the output position of the system at (*k*+1)^th^ iteration, *z*_*k*+1_ is the measurement signal at the (*k*+1)^th^ iteration, *e*_*k*+1_ is the position error of (*k*+1)^th^ iteration, *η*_*k*+1_ denotes the disturbance signal.

Before analyzing the system, two definitions need to be made. The reference tracking error is defined as:

$$e_{k}(t)=y_{d}(t)-z_{k}(t)$$ (18)

The measurement error *ϵ*_*k*_(*t*) is defined as:

$$\epsilon_{k}(t)=y_{d}(t)-y_{k}(t)$$ (19)

One can conclude from Fig 1 that $y_{k}(t)=z_{k}(t)-\eta_{k}(t)$ and combining Eqs (18) and (19) yields

$$e_{k}(t)=\epsilon_{k}(t)-\eta_{k}(t)$$ (20)

$${\dot{e}}_{k}(t)={\dot{\epsilon }}_{k}(t)-{\dot{\eta }}_{k}(t)$$ (21)

The traditional PD-ILC law is formulated as:

$$u_{k+1}^{0}(t)=u_{k}^{0}(t)+K_{1}e_{k+1}(t)+K_{2}{\dot{e}}_{k+1}(\iota )$$ (22)

where *K*_1_ and *K*_2_ are the constant learning gains of the controller. The APD-ILC law with adaptive learning gains is designed as:

$$u_{k+1}(t)=u_{k}(t)+f(e_{k+1}(t))[K_{3}e_{k+1}(t)+K_{4}e^{\beta t}e_{k+1}(t)]$$ (23)

where *e*^*βt*^ is an exponential variable gain with 0<*β*≤1, *f*(.) is a nonlinear function related to the error boundary *α* and changes with the size of the error as:

$$f(e_{k})=\{\begin{array}{ll} 1-\frac{\alpha }{\left|e_{k}\right|} & \left(|e_{k}|>\alpha \right)\\ 0 & \left(|e_{k}|\leq \alpha \right) \end{array}$$ (24)

**Fig 4:**
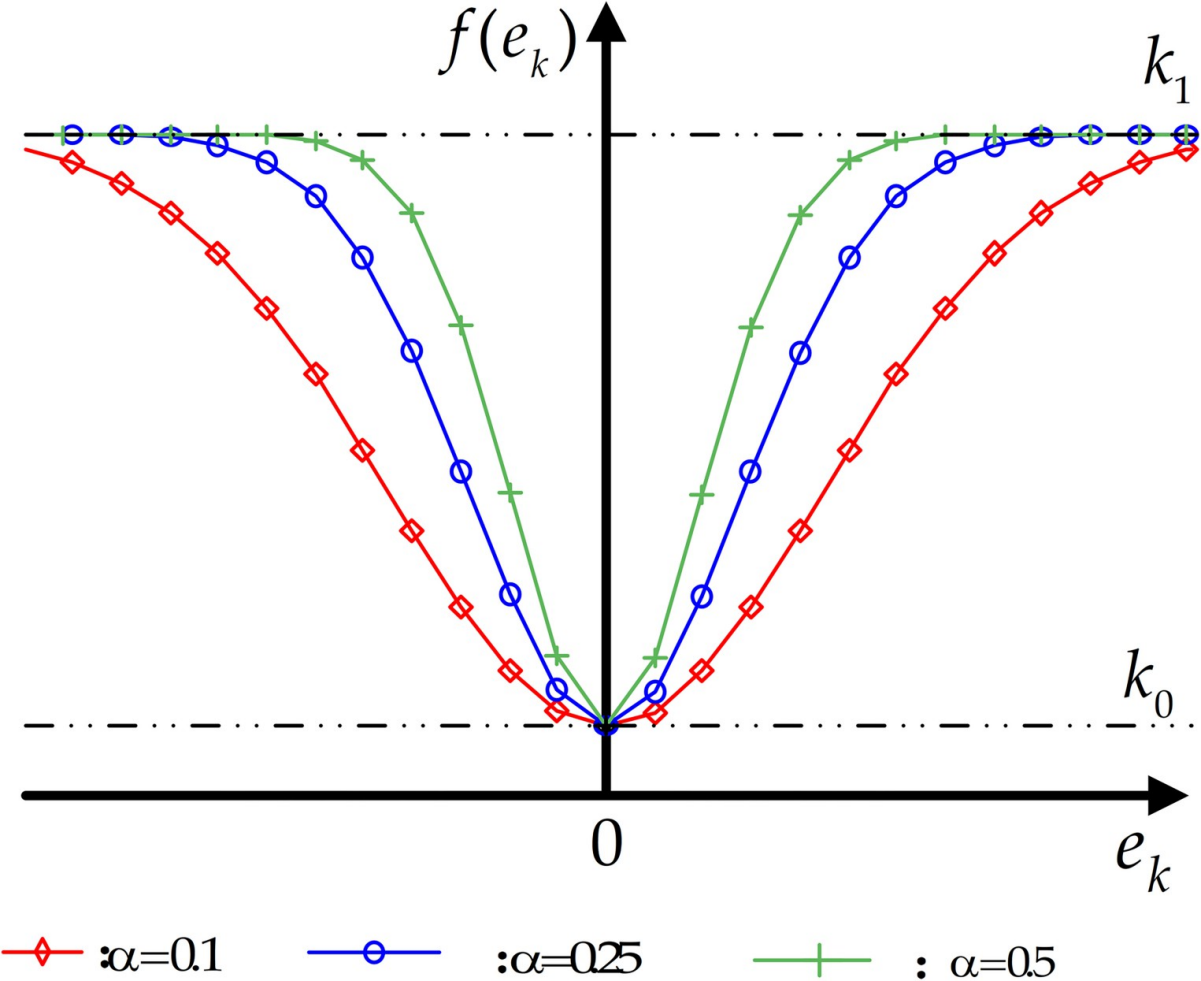
Adaptive gain function.

The resulting curve of the nonlinear error function in Eq (24) is shown in Fig 4.

**Remark 1:** For the PD-ILC with adaptive learning gains in Eq (22), it is clear that, for ∀*k*∈{0,1,2,⋯},

$$\{\begin{array}{l} K_{1,k}\in [\tau_{P}k_{0},\tau_{P}k_{1}]\\ K_{2,k}\in [0,\tau_{D}k_{1}] \end{array}$$ (25)

where *τ*_*p*_ and *τ*_*d*_ are the proportional and derivative time constants, *k*_0_ and *k*_1_ are the lower and upper limit scaling factors of the controller gains.

**Remark 2:** The selection principle of parameter *α* is as follows: A larger *α* leads to *f*(.) with a larger opening concavity, so that a small amplitude of error *e*_*k*_ can lead to a small value of *f*(.). Therefore, one should determine the value of *α* according to the amplitude of the random measurement noise, i.e. an error with a small amplitude needs a large *α* to suppress [60].

The effect of different parameters on the shape of the nonlinear function *f*(.) and the relationship between *f*(.) and the error *e*_*k*_ can be seen in Fig 4. As *α* increases from 0.1 to 0. 5, the *f*(*e*_*k*_) function reaches its maximum determined by the upper limit *k*_1_. It shows that the learning gain can be adaptively adjusted for the small errors as well as the large errors. The three curves in Fig 4 an show that the error is intuitively adjusted and compensated with the exponential gain function.

**Fig 5:**
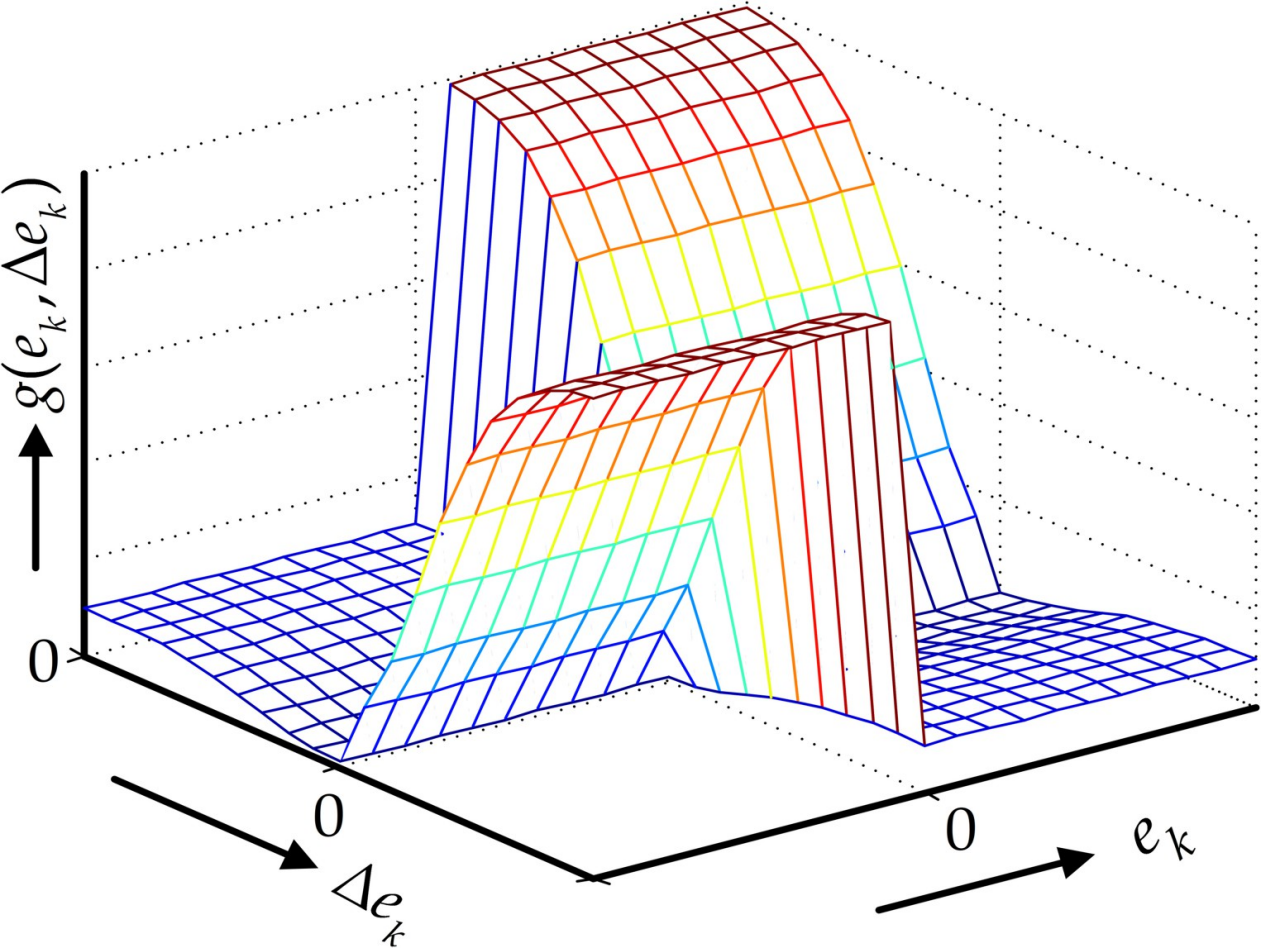
Adaptive nonlinear gain function *g*(.) versus the sample differenced error Δ*e*_*k*_.

The typical nonlinear function *g*(*e*_*k*_, Δ*e*_*k*_) versus its sample differenced error Δ*e*_*k*_ are shown in Fig 5.

In Fig 5, the sample difference error Δ*e*_*k*_ has consistent error signs during the learning process of the APD-ILC parameters. This shows that the learning process is smooth due to continuous nature of the control problem and occurs in a stable state region without chattering around the controller operating points.

Changing the damping of the controller or differential learning gain (*K*_1_) improves the adjustment speed and bandwidth of the system. It is stated that when the system error and its differential gains are different, the gains tend to reduce the tracking error. However, when the error and its rate of change are of the same sign, the value of the differential gain is increased which can improve the speed of error convergence and also leads to more stable system. Therefore, to remove the disturbance signal while retaining the useful signal, *α* should be selected to be smaller than the measurement disturbance amplitude and smaller than the error value of the amplitude.

## 4. Simulation results and discussions

Three control algorithms are simulated in MATLAB/Simulink environment to validate the effect of friction compensation proposed in this paper. These algorithms are the conventional PID control without feedforward compensation, P-ILC and APD-ILC algorithms. The parameters of the PMSM considered in this paper are given in Table 3.

**Table 3:** Parameters of the PMSM.

Parameter	Symbol	Value	Unit
Viscous friction coefficient	*B*_*m*_	0.003	*N*.*m*.*s*
Electromagnetic torque	*T*_*e*_	10	*N*.*m*
Angular velocity	*ω*(*t*)	3000	*rad*/*s*
Motor rotor inertia	*J*	1.7810^−4^	Kgm2
Armature winding inductance	*L*_*d*_ = *L*_*q*_	4	*mH*
Armature winding resistance	*R*_*s*_	1.74	Ω
Number of pole pairs	*n*_*p*_	4	-
Permanent magnet flux linkage	*ψ*_f_	0.402	Wb

The controller and filter parameters are given in Table 4.

**Table 4:** Controller and filter parameters.

Element	Description
	Limit: -15A
Current loop	PI Controller
	Proportional gain = 50
	Integral gain = 2500
	PI Controller
Speed loop	Proportional gain = 1
	Integral gain = 10
Position loop	P Controller
	Proportional gain = 200
ILC filter	Time constant t = 0.001 sec
Reference position signal	*y*_*d*_(*t*) = sin(*t*) rad

**Fig 6:**
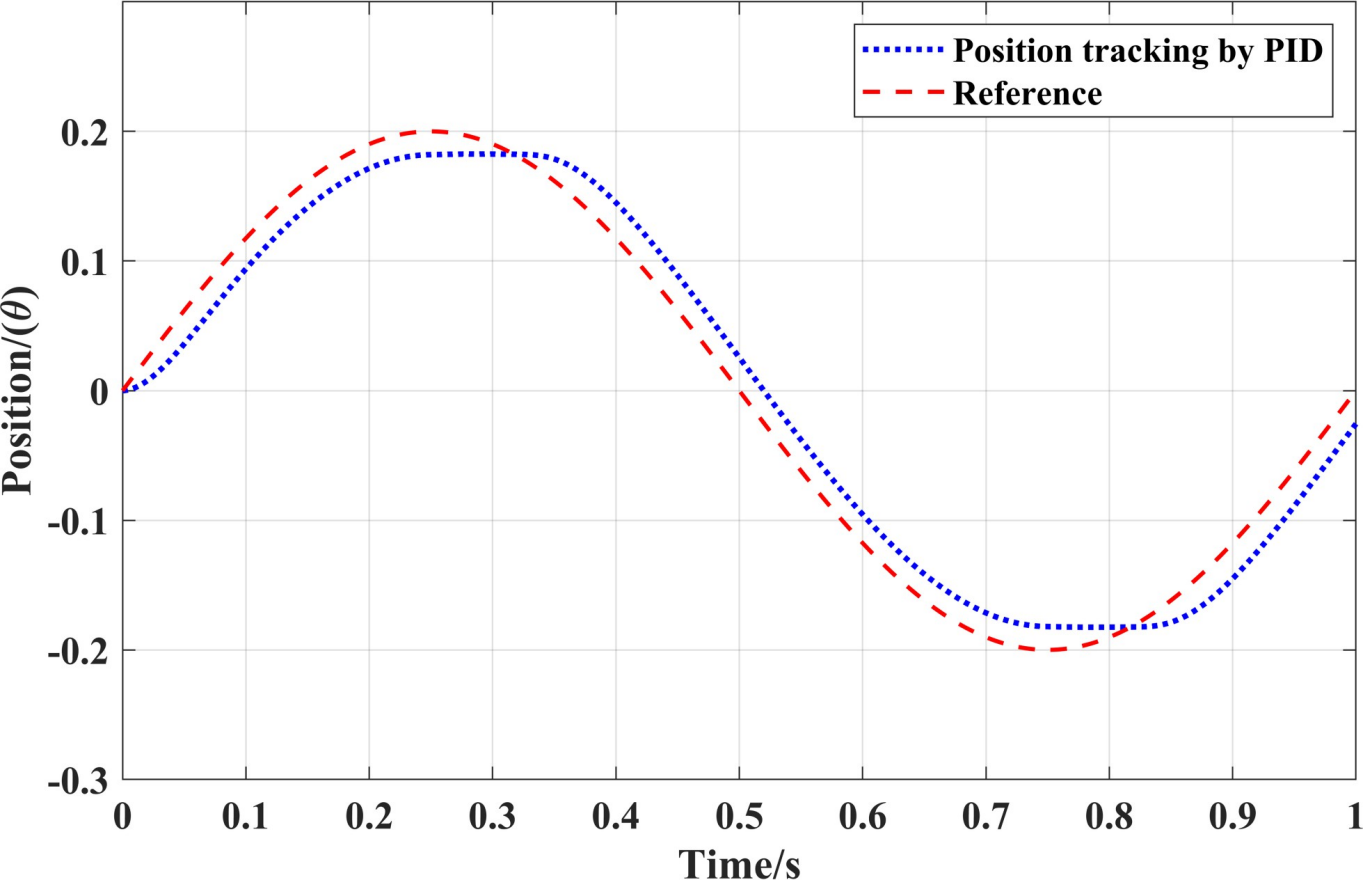
Reference position tracking with the conventional PID controller.

**Fig 7:**
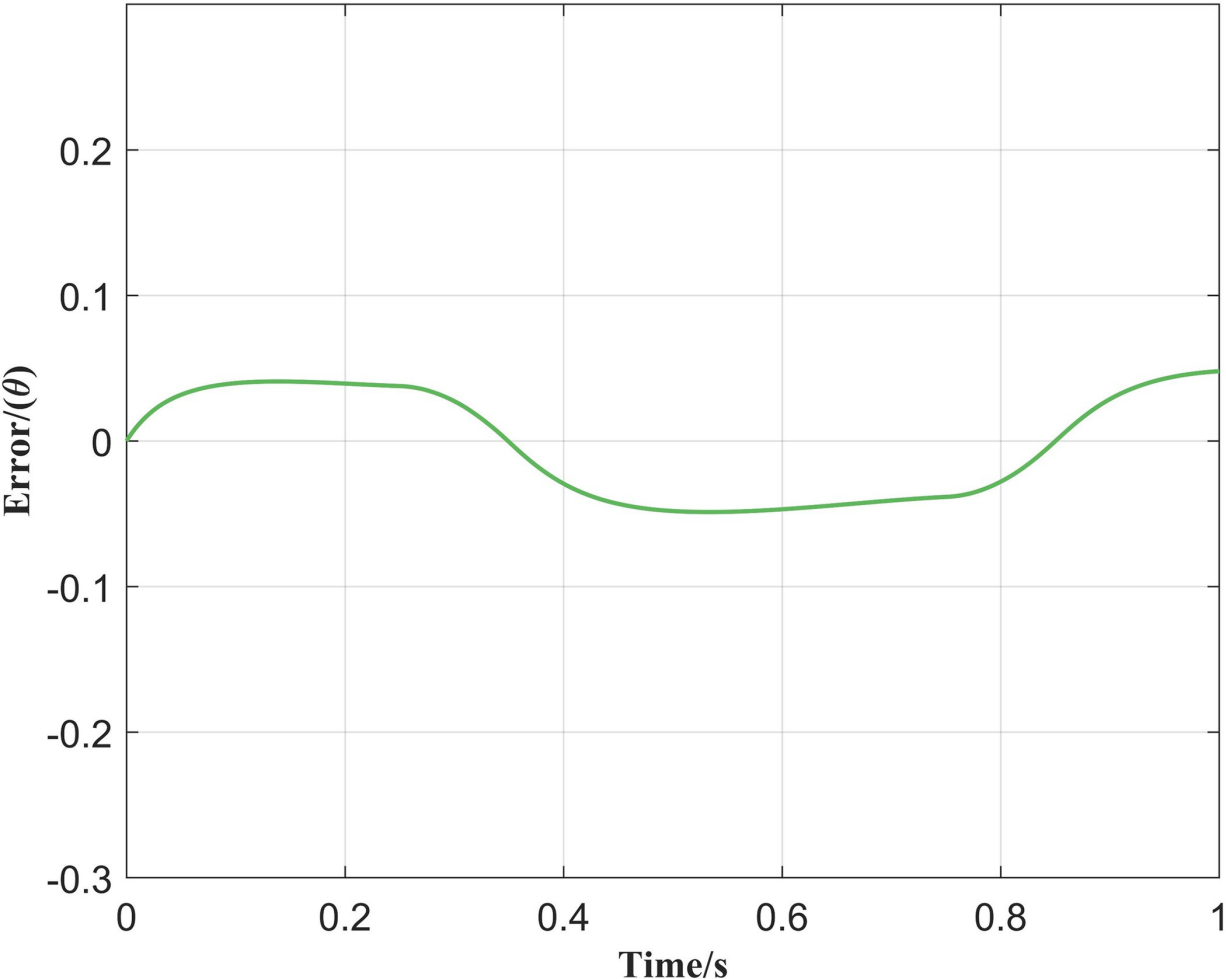
Corresponding position tracking error.

Fig 6 illustrates the position trajectory generated with the conventional PID controller and Fig 7 demonstrates the corresponding reference tracking error. It is clear that there is a bias between the reference trajectory and trajectory produced by the conventional PID controller. Therefore, the controller is unable to track the desired trajectories.

**Fig 8:**
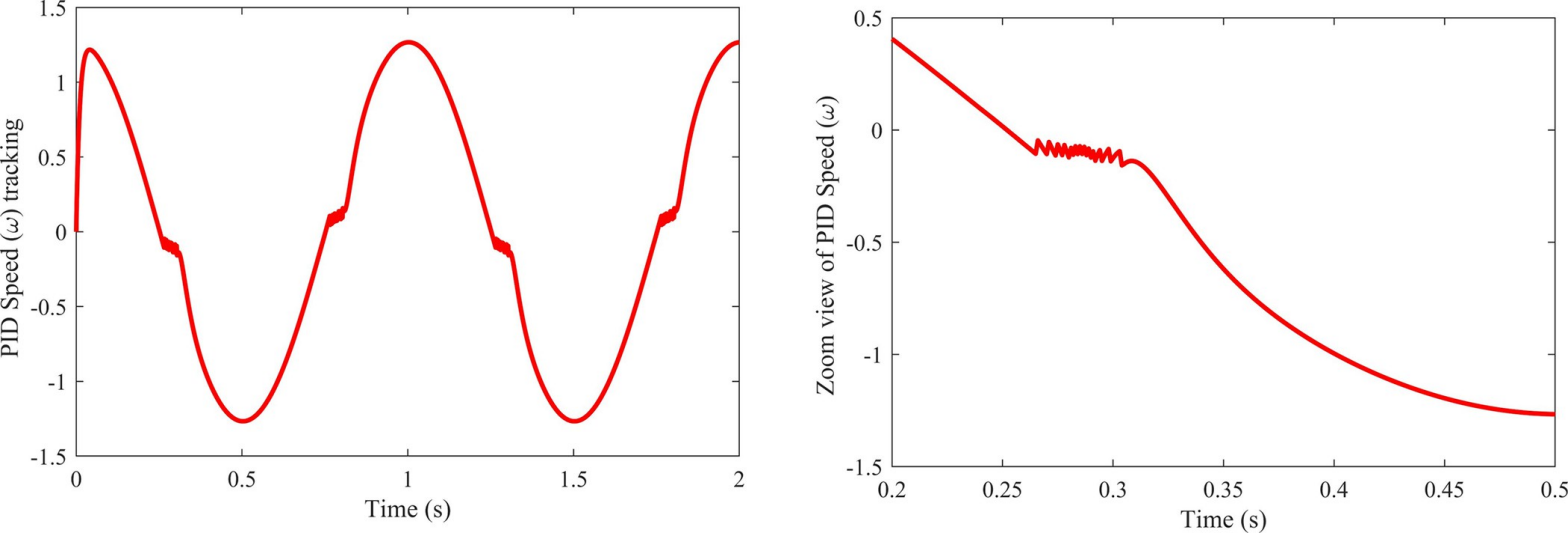
Speed trajectory with the conventional PID controller, left side shows whole speed trajectory, and right side shows partial speed trajectory with the Stribeck stemmed disturbance.

As can be seen from Fig 8, when the speed approaches zero, there is a static friction dominance whose change is multi-valued and discontinuous. This results in a noisy speed trajectory effected by the ‘Stribeck’ friction. The noise base fluctuations harm the PMSM and henceforth, they need to be compensated by the controller. However, the conventional PID controller is unable to handle such disturbances and therefore, this problem should be addressed by the adaptive control approaches.

**Fig 9:**
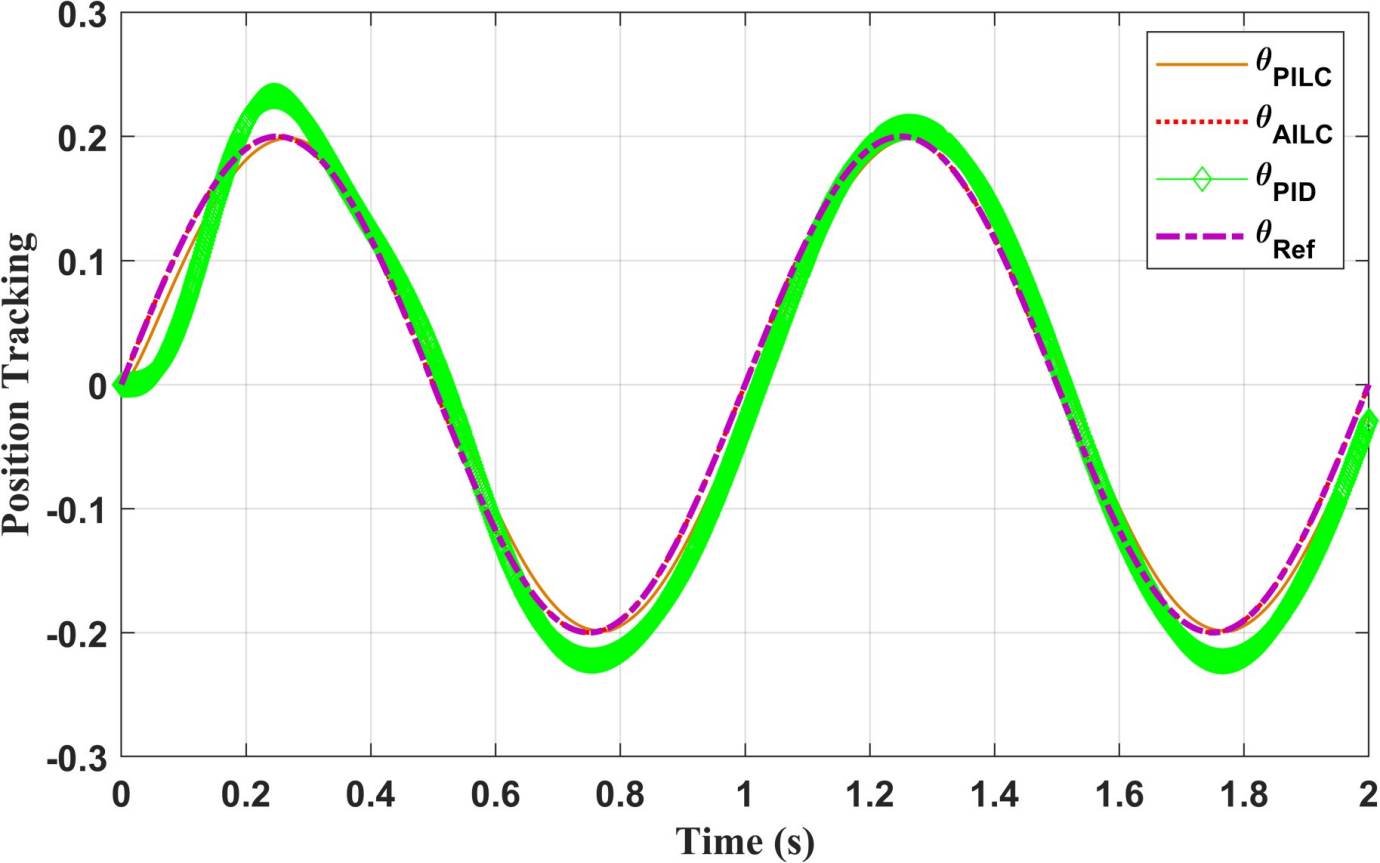
Performance comparison of the position tracking controllers.

**Fig 10:**
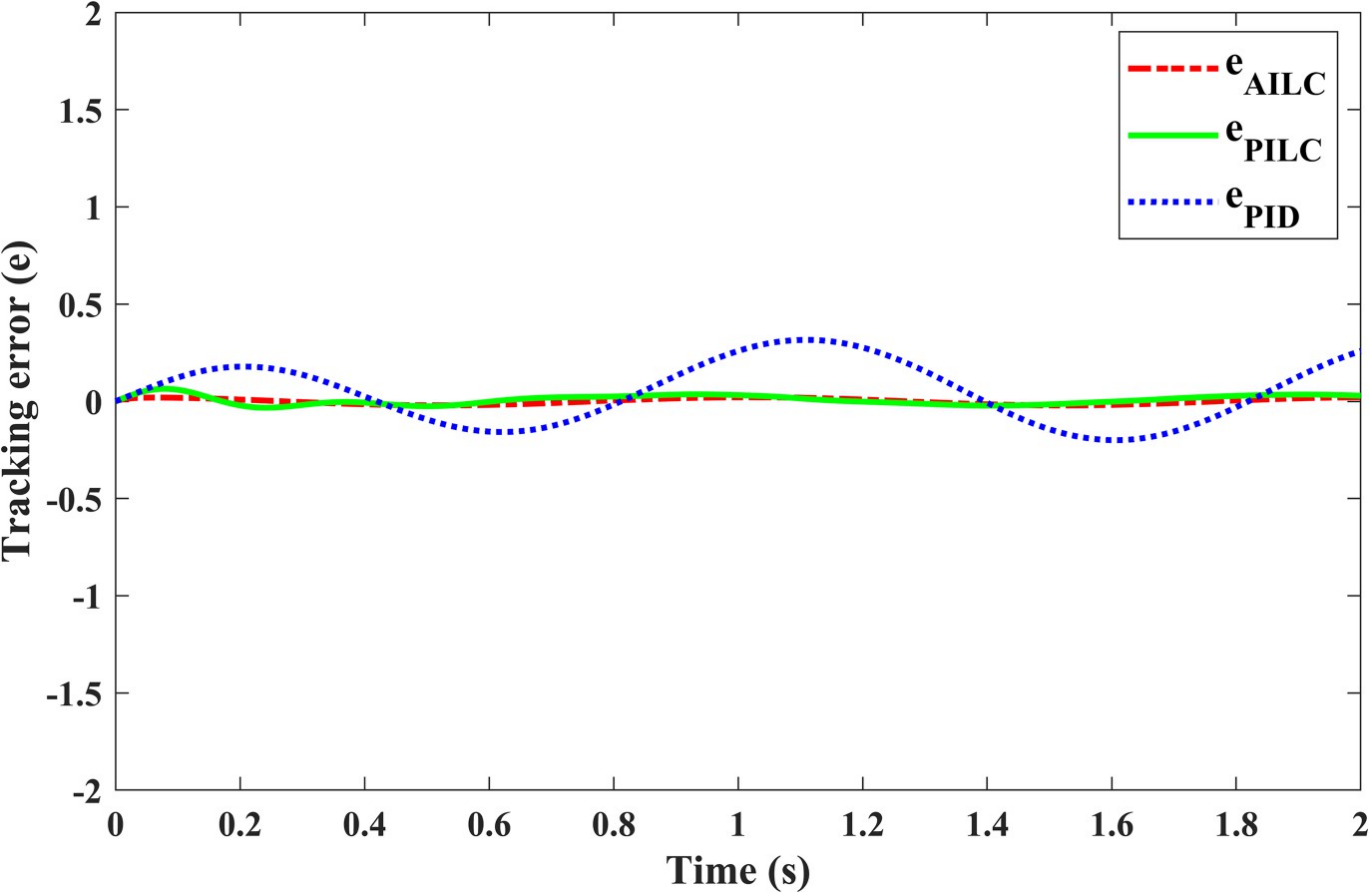
Corresponding error trajectories.

We can compare the results of the P-ILC, PID and the proposed APD-ILC algorithms as in Fig 9. It is clear from the results that the conventional PID cannot accurately track the desired position. Even though the conventional P-ILC algorithm attempts to track the desired position, it eventually yields a noticeable tracking error. Thus, the error cannot be eliminated completely with the P-ILC as well. On the contrary, APD-ILC algorithm closely tracks the desired position and eliminates the friction effect to a large extent. Fig 10 shows the tracking errors where the error convergence with APD-ILC is superior since the it ultimately converges to zero, while the errors in other controllers (PID, P-ILC) remain non-zero.

**Fig 11:**
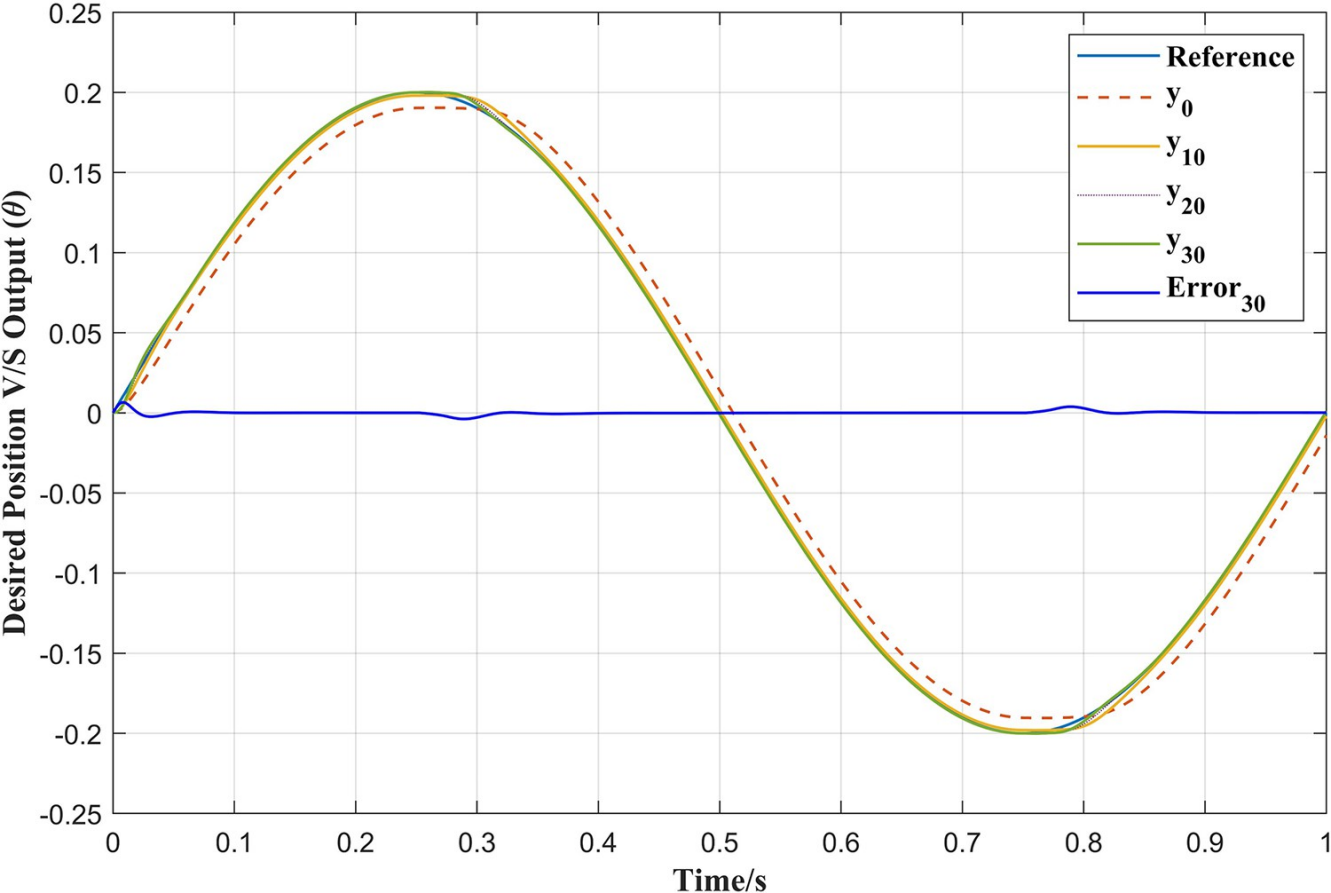
APD-ILC position tracking.

**Fig 12:**
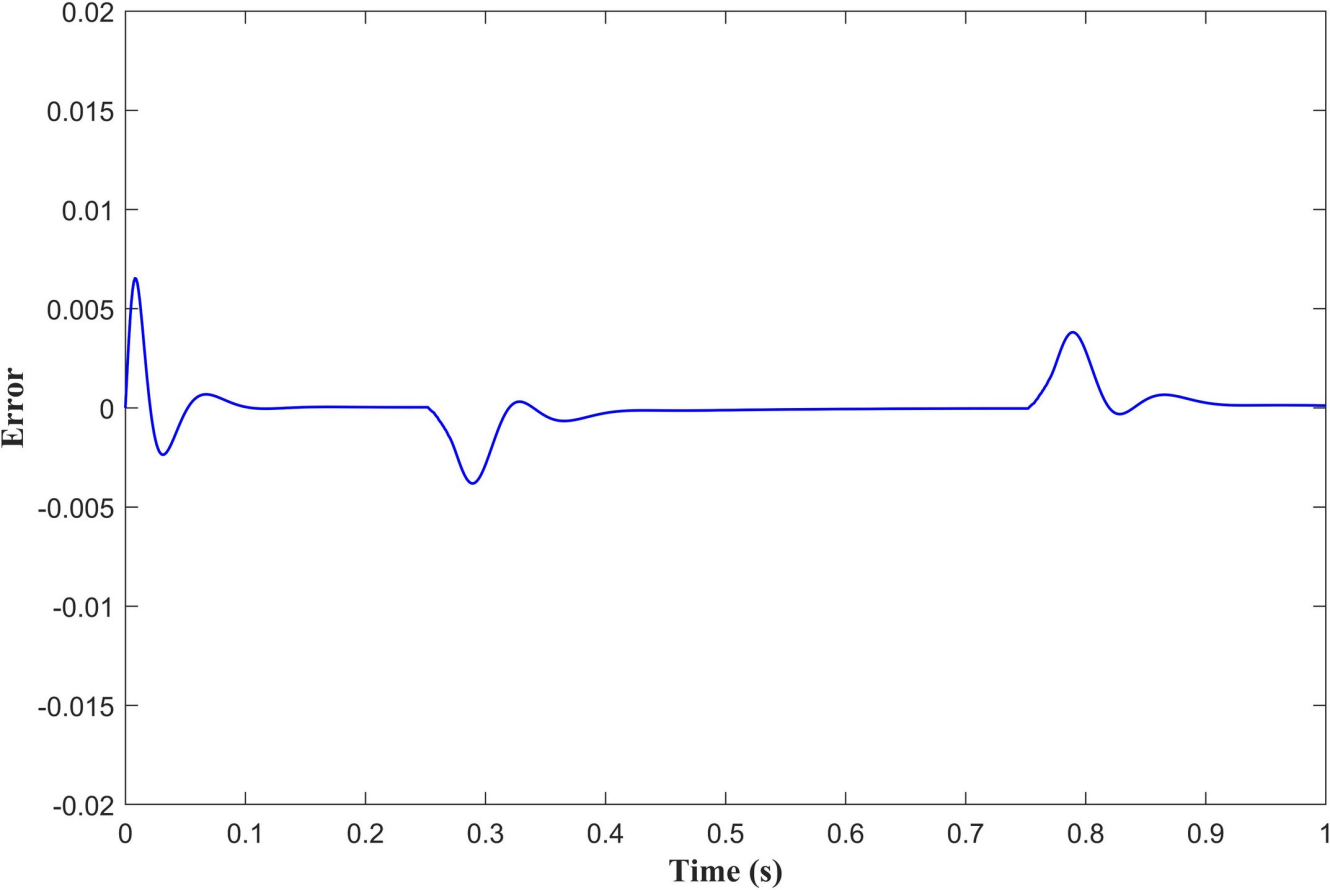
APD-ILC position tracking error.

Fig 11 shows the position tracking results with the APD-ILC for different iterations. It is clear that the tracking for 30^th^ iteration *y*_30_ is more accurate than the 0^th^ iteration *y*_0_, 10^th^ iteration *y*_10_ and 20^th^ iteration *y*_20_. Therefore, it can be stated that the tracking accuracy improves as the number of the iterations increase. It can be also noticed that the proposed APD-ILC offers proper friction compensation in the position tracking. The position tracking error curve is illustrated in Fig 12 also confirms the position tracking efficiency.

**Fig 13:**
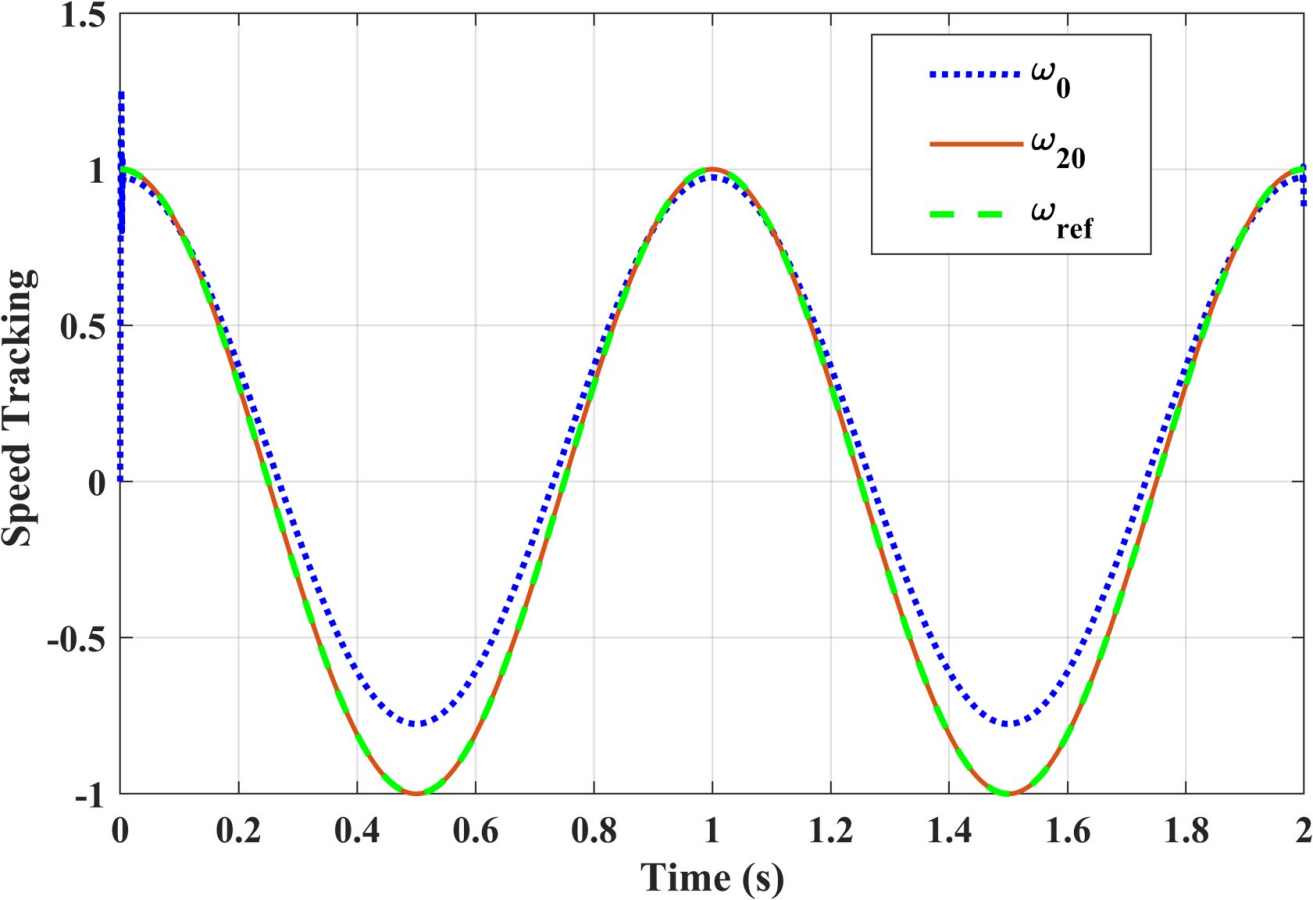
APD-ILC speed trajectories for different iterations.

Fig 13 presents the speed tracking results for two different iterations; *ω*_0_ is 0^th^ iteration, *ω*_20_ is 20^th^ iteration with respect to the reference speed *ω*_*ref*_. It can be concluded from the figure that the friction compensation strategy dramatically improves the speed trajectory in the presence of the Stribeck friction.

Table 5 shows the position tracking errors for the P-ILC, PD-ILC and APD-ILC algorithms. It can be noticed that the proposed APD-ILC yields tracking error almost the half of the other control algorithms. After 20^th^ iterations, the errors of P-ILC and PD-ILC algorithms produce 0.175381 and 0.024335 errors, respectively. It is clear that all the tracking errors reduce consecutively with the number of iterations increases. This occurs because all these control algorithms learn the unknown control parameters iteratively. The tracking error of the proposed APD-ILC algorithm is the smallest as compared to the other two control algorithms. Therefore, it can be easily observed from the Table 5 that the convergence speed of the proposed APD-ILC algorithm in this paper has significantly higher accuracy compared to other traditional control algorithms.

**Table 5:** Comparative values of tracking errors corresponding to ILC laws.

Iteration(k)	P-ILC	PD-ILC	APD-ILC
1^st^	7.21731636	7.11731545	3.28217325
5^th^	3.049819	2.598041	1.232084
10^th^	0.953581	0.584192	0. 29215
15^th^	0.375380	0.243351	0.003683
20^th^	0.175381	0.024335	0.001543

**Fig 14:**
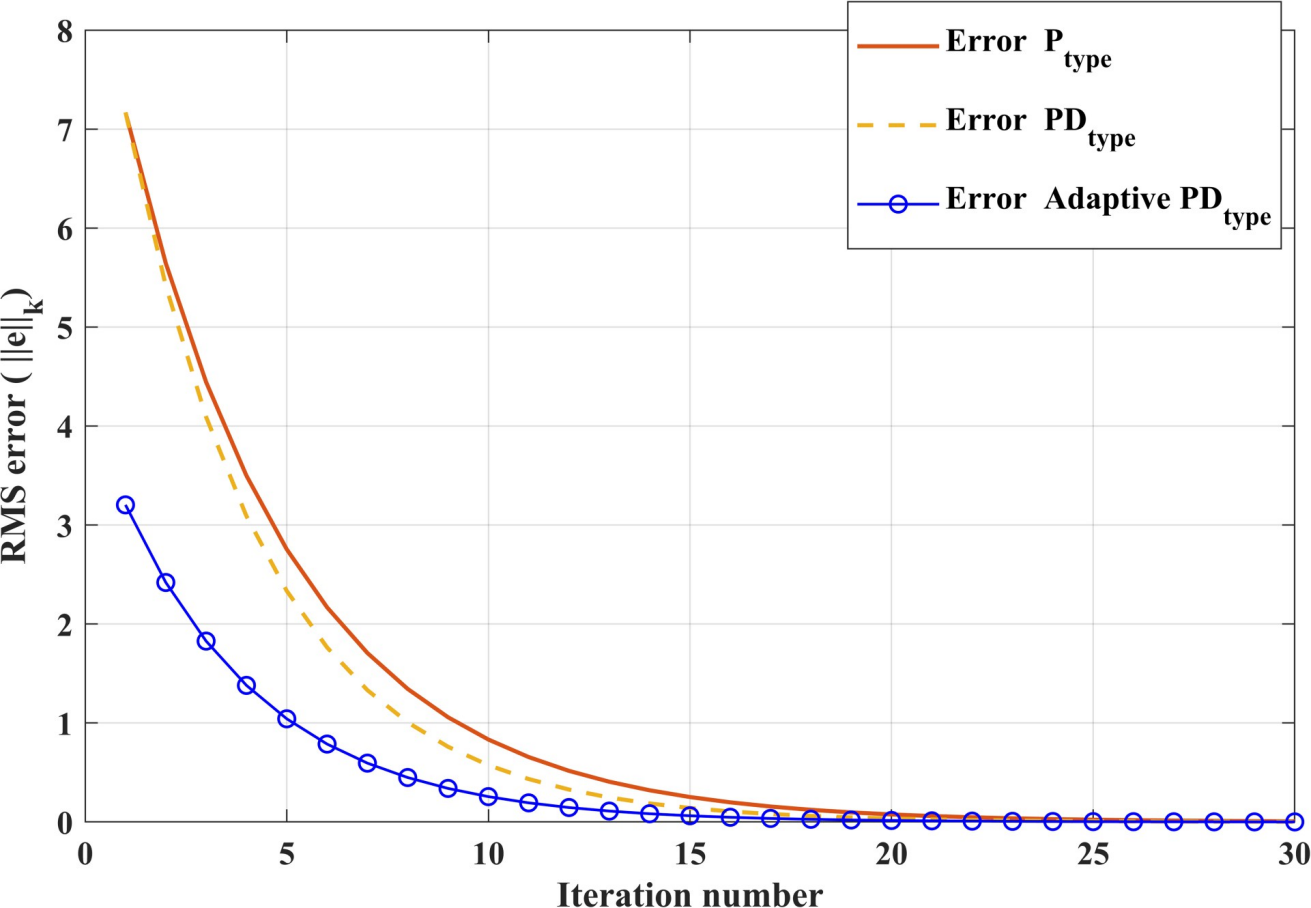
RMS error of the controllers for 30 iterations.

The position tracking error trajectories for the P-ILC, PD-ILC and APD-ILC algorithms are shown in Fig 14 with respect to the number of iterations. Due to the adaptive property of the APD-ILC algorithm, its RMS tracking error reduces quicker through the 30 iterations. Eventually, the tracking error converges to zero with increasing number of iterations which indicates that the proposed controller is efficient in dealing with motor nonlinearities and the Stribeck friction, particularly in servo control applications.

## 5. Conclusion

This paper proposed the APD-ILC algorithm to handle the Stribeck friction problem in the PMSM servo system. The genetic algorithm has been used to identify the nonlinear Stribeck friction unknown parameters with the offline training data and the constructed friction model was then used for the feedforward compensation. Finally, the APD-ILC algorithm has been designed to compensate the predicted Stribeck friction and also to track the desired position trajectory. An extensive comparison analyses has been performed to show the effectiveness and reliability of the proposed adaptive method. It was revealed that the conventional PID, P-ILC, PD-ILC algorithms were unable to overcome the friction disturbance properly, whereas the proposed APD-ILC was able consider the Stribeck friction and also eliminate it as desired. Thus, the proposed APD-ILC algorithm is robust and efficient to deal with the nonlinearities compared to the conventional control approaches. Other nonlinearities such as the parameter uncertainties in the servo system, gears backlash, and combined friction nonlinearity with external load disturbances can be considered in future research.
